# Hormone Receptor Signaling and Breast Cancer Resistance to Anti-Tumor Immunity

**DOI:** 10.3390/ijms242015048

**Published:** 2023-10-10

**Authors:** Alexandra Moisand, Mathilde Madéry, Thomas Boyer, Charlotte Domblides, Céline Blaye, Nicolas Larmonier

**Affiliations:** 1 CNRS UMR 5164, ImmunoConcEpT, Biological and Medical Sciences Department, University of Bordeaux, 33076 Bordeaux, France; alexandra.moisand@u-bordeaux.fr (A.M.); mathilde.madery@u-bordeaux.fr (M.M.); thomas.boyer@u-bordeaux.fr (T.B.); charlotte.domblides@chu-bordeaux.fr (C.D.); 2 Cancer Biology Graduate Program, UB Grad 2.0, University of Bordeaux, 33076 Bordeaux, France; 3 Department of Medical Oncology, University Hospital of Bordeaux, 33000 Bordeaux, France

**Keywords:** hormone receptors, estrogen receptors, hormone signaling, breast cancers, cancer immunity, immunotherapies

## Abstract

Breast cancers regroup many heterogeneous diseases unevenly responding to currently available therapies. Approximately 70–80% of breast cancers express hormone (estrogen or progesterone) receptors. Patients with these hormone-dependent breast malignancies benefit from therapies targeting endocrine pathways. Nevertheless, metastatic disease remains a major challenge despite available treatments, and relapses frequently ensue. By improving patient survival and quality of life, cancer immunotherapies have sparked considerable enthusiasm and hope in the last decade but have led to only limited success in breast cancers. In addition, only patients with hormone-independent breast cancers seem to benefit from these immune-based approaches. The present review examines and discusses the current literature related to the role of hormone receptor signaling (specifically, an estrogen receptor) and the impact of its modulation on the sensitivity of breast cancer cells to the effector mechanisms of anti-tumor immune responses and on the capability of breast cancers to escape from protective anti-cancer immunity. Future research prospects related to the possibility of promoting the efficacy of immune-based interventions using hormone therapy agents are considered.

## 1. Introduction

**Figure 1:**
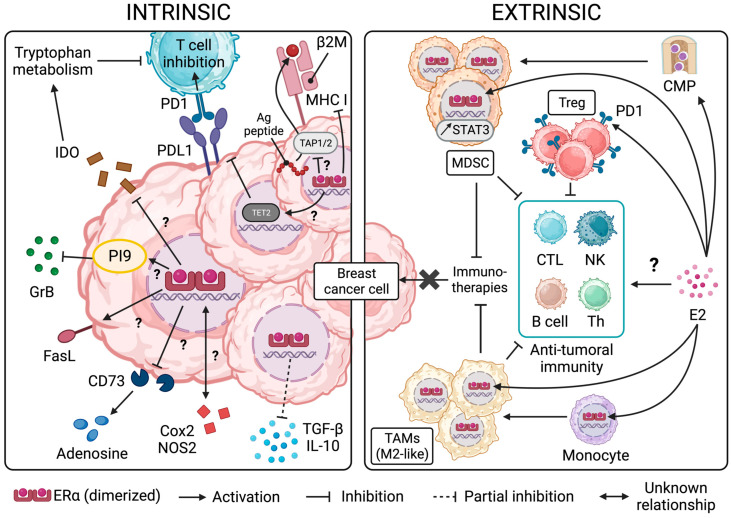
The impact of ER signaling on the intrinsic and extrinsic mechanisms of the escape of BC cells from anti-cancer immunity. An overview of current knowledge related to the impact of estrogen signaling on the mechanisms of escape from effector anti-tumoral immune cells and immunotherapies. It should be noted that most of the studies to date have focused on estrogen signaling through ERα. MHC I, major histocompatibility complex class I; β2M, light chain β-2-microglobulin; TAP, transporter associated with antigen processing; TET2, ten-eleven translocation 2; PDL1, programmed-death ligand 1; PI9, serpinB9/proteinase inhibitor 9, GrB, granzyme B; IDO, indoleamine 2,3-dioxygenase; FasL, Fas ligand; Cox2, cyclooxygenase 2; NOS2, nitric oxide synthase 2; TGF-β, transforming growth factor β; MDSC, myeloid-derived suppressor cell; CMP, common myeloid progenitor; T_reg_, regulatory T cell; PD1, programmed cell death 1; CTL, cytotoxic T lymphocyte; NK, natural killer cell; Th, helper T lymphocyte; E_2_, 17-β estradiol; TAMs, tumor-associated macrophages; STAT3, signal transducer and activator of transcription 3. The question mark represents mechanisms that are controversial or need to be addressed. Illustration created using BioRender.

The advent of immunotherapies over the last decade has constituted a major breakthrough in the treatment of cancer patients. However, although some patients with specific types of cancers, such as lung, bladder, colon, and liver malignancies, have benefited from such immune-based therapies, breast cancer (BC) patients have yet to experience comparable advantages. This relative absence of response to efficient immune-based interventions may be related to the multiple mechanisms of escape triggered by developing cancers. These mechanisms of resistance to immunotherapies can be “tumor-intrinsic” (the upregulation of ligands binding to inhibitory immune checkpoint receptors on effector immune cells and the impaired expression and presentation of neo-antigens, for instance) or “extrinsic”, thereby including non-malignant cells of the tumor microenvironment (TME), such as immunosuppressive immune cells and stromal cells (Figure 1). These interacting networks of intrinsic and extrinsic escape mechanisms are particularly efficient in BC to avoid immune-mediated elimination and sustain primary tumor growth and malignant cell invasion and metastasis [1,2,3,4].

Breast cancers regroup many heterogeneous diseases with different prognoses and sensibilities to treatment and can be categorized by their receptor expression: hormone receptors (HRs), namely the estrogen (ER) and progesterone (PR) receptors, and the human epidermal growth factor receptor 2 (HER2) [5]. This heterogeneity was further unraveled by the molecular classification proposed by Perou et al. and Sørlie et al., who classified breast cancers into four groups: luminal breast cancer (characterized by the expression of *ESR1*, coding for ERα), HER2-enriched, basal-like breast cancers, and the controversial “normal breast-like” [6,7]. In clinical practice, a simplified classification is used based on the St Gallen International Expert Consensus. Luminal breast cancers are defined by their positivity for the ER receptor and can be further distinguished into “luminal A” and “luminal B” breast cancers. In the molecular classification, “luminal B” breast cancers display a lower expression of *ESR1* and a higher expression of proliferation genes. In the simplified classification, “luminal A” breast cancers have a high ER and PR expression (PR ≥ 20%), a low Ki67 index, and no amplification of HER2. Conversely, “luminal B” breast cancers are generally more aggressive, with a PR ≤ 20% and a higher Ki67 index. This category also comprises “luminal B- non HER2” or “luminal B- HER2-enriched”. “HER2-enriched” breast cancers are characterized by an amplification of HER2, and basal-like breast cancers correspond roughly to triple-negative breast cancers, which are HR^−^/HER2^−^ [8,9,10,11]. Further characterization of their tumor microenvironment (TME) reveals that these subtypes also differ in the composition of non-malignant elements, which may significantly impact responses to immunotherapies [12]. As such, TNBC patients, whose tumors are considered to be more immunogenic, are more responsive to an immune checkpoint blockade (ICB), such as immune inhibitory receptor PD1 inhibition [13,14]. On the other hand, hormone-dependent BCs, which comprise around 70% of all BCs, are much less sensitive to ICBs, which is an observation closely related to a more moderate mutational load of tumors, a reduced tumor-infiltrating lymphocyte (TILs) number, and sporadic PDL1 (programmed-death ligand 1, a ligand of PD1) expression [15]. Recent studies have revealed that the TME associated with HR^+^ tumors is largely composed of immune cells, such as myeloid cells and T lymphocytes, which may participate in immunotherapy resistance [16,17,18].

In the current article, we review and discuss the potential role of HR and, more specifically, ER signaling in the resistance to the effector mechanisms of protective anti-tumor immunity and immune-based therapies. The state of knowledge related to the impact of currently available anti-hormonal treatments on breast cancer sensitivity to immune effector mechanisms is discussed. Points of current controversies and areas lacking scientific knowledge in the field are identified, and future research prospects related to the role of HR signaling and its modulation on immune responses and immunotherapies in breast cancers are considered. There is a specific emphasis placed on the oncogenic driver ER, as it holds a major role in the development of HR^+^ breast tumors.

## 2. The Estrogen-ER Pathway in BC

### 2.1. Overview of Estrogen-ER Signalings

**Figure 2:**
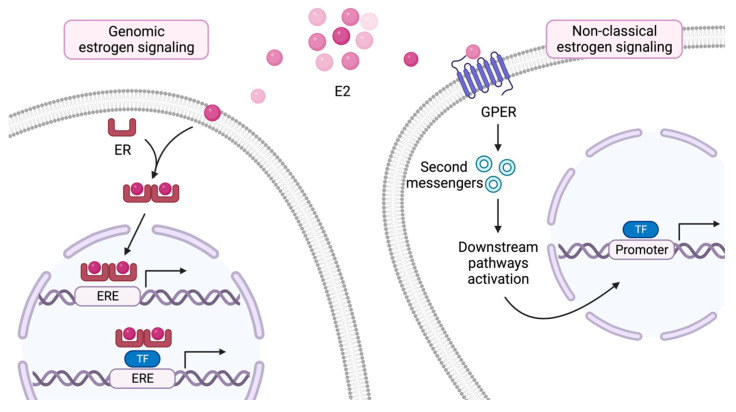
A simplified overview of estrogen-induced signaling pathways. In the classical genomic signaling pathway, estrogens diffuse through the cell membrane and bind to estrogen receptors, inducing their dimerization. ER complexes translocate to the nucleus where they directly or indirectly (through the interaction with co-activating factors) interact with EREs to activate the transcription of downstream genes. On the other hand, gene transcription associated with the non-classical estrogen signaling pathway is characterized by the activity of an estrogen-bound GPER, without the involvement of a cytosolic ER. E_2_, 17-β estradiol; ER, estrogen receptor; GPER, G protein-coupled estrogen receptor; ERE, estrogen response element; TF, transcription factor. Illustration created using BioRender.

Estrogens are steroid hormones, and the most common in humans is 17β-estradiol (E_2_), which plays a dualistic role in physiological and pathological circumstances. Estrogen signaling is mediated by two major pathways. The genomic or “classical” signaling pathway involves estrogen receptors (ERs), acting as nuclear transcription factors, and “non-classical” alternatives, relying primarily on the activity of the membrane-anchored G protein-coupled estrogen receptor 1 (GPER1) [5,19]. In the classical pathway, the binding of estrogens to ER isoforms ERα and/or ERβ allows the translocation of dimerized complexes of the receptors to the cell nucleus, wherein further binding to estrogen-response elements (EREs) activates or represses the transcription of target genes controlling the regulation of the cell cycle, proliferation, and apoptosis [5,19,20] (Figure 2).

In the setting of luminal BC, the dysregulation of ER signaling plays a major role in carcinogenesis and tumor progression. In this context, ERα has been demonstrated to be critically involved in these processes [21]. The treatment of patients with luminal BC has thus largely aimed at blocking the estrogen/ER axis, either by inhibiting the activity of the aromatase enzyme to limit endogenous production of estrogens or by directly antagonizing the ER [20,22]. In contrast with these observations, the activation of ERβ in BC cells may be associated with more favorable outcomes, notably by impairing tumor cell growth and inducing apoptosis [23]. However, the precise mechanisms associated with this anti-proliferative effect remain to be determined.

### 2.2. Targeting of the Estrogen-ER Pathway

Two main categories of ER-targeting agents have been developed, which differ by their mode of action: selective estrogen receptor modulators (SERMs) and selective estrogen receptor degraders (SERDs). The former acts by modifying ER transcriptional complexes through the recruitment of corepressors, leading to the silencing of ER target genes [20]. Tamoxifen is an example of a widely administered SERM that has proven highly effective in the treatment of early ER^+^ BC. This agent may also be administered in advanced settings under specific conditions [20,24]. Nevertheless, resistance to tamoxifen is frequent, and its prolonged use could increase the risk of endometrial cancer [25,26]. In this context, SERDs, such as fulvestrant, have been developed with the unique ability to prevent ER activation by binding to an ER in its monomeric form and inducing its degradation through the ubiquitin–proteasome pathway [20]. Contrar to tamoxifen, SERDs are considered “pure” ER antagonists due to their lack of agonistic effects in tissues other than the breast [20].

It is noteworthy that although the estrogen-ER axis in BC cells remains the most widely studied mechanism of tumorigenesis promoted by estrogens, these hormones may also exhibit effects on non-malignant cells of the tumor microenvironment (TME), indirectly fostering tumor growth, as will be discussed in Section 3.

## 3. ER Signaling and Resistance/Sensitivity of BC Cells to Effector Immune Cells and Immunotherapies

### 3.1. The Role of ER Signaling in Malignant Cell Resistance to Anti-Tumoral Effectors

Effector anti-tumoral CD8^+^ T lymphocytes (cytotoxic T cells, CTL) recognize tumor-specific antigens in the form of processed peptides associated with the major histocompatibility complex class I (MHC I) molecules expressed at the surface of cancer cells. The downregulation of MHC I expression or deficiencies in the antigen-presenting machinery in tumor cells represents a major mechanism of escape from immune recognition and thus elimination.

*The impact of ER signaling on antigen presentation in MHC Class I*

To date, information related to the impact of ER signaling on antigen presentation on MHC I in BC remains sparse and controversial. Although initial observations by Rodríguez et al. [27] identified 17-β estradiol as an enhancer of HLA-I (human MHC class I) expression in ER^+^ (MCF-7) but not in ER^−^ (MDA-MB-231 and MDA-MB-435s) BC cell lines, the underlying mechanism has yet to be identified. Furthermore, the nature of the antigens presented by MHC I complexes was not investigated. A recent analysis of gene expression data from 59 BC cell lines retrieved from the CCLE (Cancer Cell Line Encyclopedia) highlighted an inverse relationship between *ESR1* gene expression and HLA genes [28]. A later study conducted by the same group uncovered a higher baseline expression of HLA-A, B, and C proteins in ER^−^ MDA-MB-231 cells compared to ER^+^ MCF-7 and T47D [29]. Consistently, treatment with the ER downregulator fulvestrant led to an increase in HLA-ABC expression in MCF-7 and T47D. However, the expression of HLA-ABC proteins in this study was not compared between MDA-MB-231 at baseline and MCF-7 and T47D post-fulvestrant treatment. On the other hand, RNA sequencing and the subsequent gene set enrichment analysis (GSEA) of MCF-7 grown in prolonged estrogen deprivation conditions or with fulvestrant showed a marked reduction in *HLA-A* and *-C*, *B2M,* and *TAP2* genes, which are all involved in the antigen-presentation machinery [30]. Although contradictory, these pre-clinical studies highlight the complexity of the potential regulation of MHC I molecules by an ER at the various levels investigated.

Recent clinical data are more straightforward and argue in favor of a negative regulation of MHC I complexes by an ER. A study of primary breast cancer tissues from patients who did not receive chemotherapy or radiotherapy clearly demonstrates a negative correlation between ER status, the expression of HLA-I genes in tumor cells, and lymphocyte infiltration in tumor sites (Figure 1). The analysis of 396 TCGA (The Cancer Genome Atlas) BC cases examined under PAM50 standards, a prediction test routinely performed following biopsy or surgery, further supports these clinical observations by confirming the negative correlation between HLA-A expression, IFN signaling (a master inducer of MHC I expression), and ER activity. In addition, HR^−^/HER2^+^ BC and TNBC were found to exhibit higher HLA-A, B, and C expression than luminal HR^+^ tumors. Whether the ERα isoform specifically impacts the expression of HLA genes in ERα^+^ luminal A compared to ERα^−^ luminal B tumors remains to be determined. In line with these results, clinicopathological data from 126 patients with ER^+^/HER2^−^ invasive ductal carcinoma treated with estrogen modulators indicated higher HLA-ABC positivity and TIL infiltration along with a lower ER Allred score (a histological quantification of ER and PR) compared to the group receiving chemotherapy alone [29].

The study of invariant light-chain β-2-microglobulin (β2M, required to maintain MHC I molecules conformation) transcripts in 166 BC samples obtained following resection from patients without previous chemotherapy and radiotherapy failed at establishing a link between BC molecular subtype or ER status and the expression of β2M messenger RNA (mRNA) [31]. However, contrasting these results, an analysis of FFPE samples from 164 BC patients following surgery indicated that β2M protein expression was significantly reduced in ER^+^ compared to ER^−^ patients [31] (Figure 1).

Altogether, these results advocate for a contributing role of ER signaling in the regulation of MHC I expression in estrogen-dependent BC (Figure 1), which may consequently impact the recognition and subsequent elimination of BC cells by CD8^+^ cytotoxic T lymphocytes. However, further studies are needed to clearly delineate the mechanistic link(s) between MHC class I expression and ER signaling in the different breast cancer subtypes. Likewise, how the observed downregulation of MHC class I may impact recognition and killing by natural killer cells (NKs) remains to be determined.

*The impact of ER signaling on the antigen-presenting machinery*

TAP1 and TAP2 (transporters associated with antigen processing 1 and 2) are essential elements of the MHC I-presenting machinery. TAP1 and TAP2 actively transfer peptides from the cytosol to the endoplasmic reticulum, where they are loaded onto MHC I molecules before transiting as complexes toward the cell surface for recognition by CD8^+^ T lymphocytes [32]. The reduced expression or mutations of TAP1 and/or TAP2 proteins in tumors result in the absence of MHC I-peptide complexes at the cell surface. This consequently leads to a lack of recognition by CTL [33,34,35,36,37,38].

As it relates to BC, only limited information is available on the possible dysregulation of TAP1 and TAP2. Vitale et al. initially observed that a downregulation of HLA I associated with both TAP1 and TAP2 reduced expression in the majority of primary high-grade BC lesions studied (68%) but not primary low-grade lesions, regardless of the molecular subtype [39] (Figure 1). In metastatic BC, the loss of TAP1 was associated with a complete loss of HLA I in both primary and metastatic tumors, while HLA I molecules were not significantly reduced in ER^+^ primary tumors [40]. In another study, lower levels of TAP2 were observed in primary tumors positive for ERs or PRs [41]. However, in contrast with these studies, a recent analysis of 160 primary BC tumors highlighted increased TAP1 expression in stage 2 compared to stage 1 tumors [42]. In this report, TAP1 and TAP2 expression was significantly higher in high-grade BC, corresponding to more aggressive, basal forms of the disease [42]. These somehow conflicting clinical observations further underscore the need for additional studies, including pre-clinical investigations, to accurately define the link between ER signaling, TAP, and MHC class I expression, antigen presentation, and the efficient elimination of BC cells by CTL.

*The impact of ER signaling on tumor cell resistance to cytotoxic immune effectors*

Tumor cells can also actively inhibit anti-tumoral immunity, notably through the blockade of cytotoxic mechanisms. CTL or NK cells can induce cell lysis through the release of perforin and granzyme-containing granules, which lead to target cell apoptosis. Tumor cells have evolved many mechanisms to resist CTL- and NK-mediated killing, including the expression of the serine protease inhibitor serpinB9/proteinase inhibitor 9 (PI-9) [43,44]. Importantly, PI-9 expression can be triggered by primary estrogens [45] (Figure 1). In the BC MCF-7 cell line, PI-9 is rapidly induced by 17-β estradiol (E_2_) and exhibits a protective effect against NK cell cytotoxicity. This result indicates that circulating amounts of estrogens (around 50 pM) found in post-menopausal women may be sufficient to counteract the killing activity of NK cells through the induction of PI-9 by BC cells [46] (Figure 1). Interestingly, it has been shown that 4-hydroxytamoxifen (OHT), the active metabolite of the SERM tamoxifen, induced a 10-fold increase in PI-9 mRNA in MCF-7 cells and exhibited comparable effects to E_2_ in inhibiting NK-mediated cytotoxic responses. Such surprising results are likely the consequence of the function of tamoxifen as an ER transcriptional complex modulator rather than a pure ER antagonist. On the other hand, excess raloxifene or fulvestrant blocked the E_2_-induction of PI-9 mRNA in MCF-7 [46]. Along these lines, MCF-7 cancer stem cells were found to express higher levels of PI-9 than their adherent/parental counterparts, and E_2_ supplementation was used to further enhance PI-9 expression in these MCF-7 CSC. This increase was concomitant to an augmentation of the Erα36 isoform known to mediate rapid non-genomic actions of estrogens, and a decrease in the nuclear Erα66 [47]. Therefore, PI-9-regulated expression may not be restricted to the classical estrogen signaling axis driven by Erα66 as initially postulated. These results, however, need to be confirmed with additional BC cell lines. In addition, prospective studies are warranted to confirm the role of PI-9 in tumor-associated immunosuppression, as well as pre-clinical and clinical investigations, to potentially develop innovative strategies targeting the activity of PI-9 in BC.

**Figure 3:**
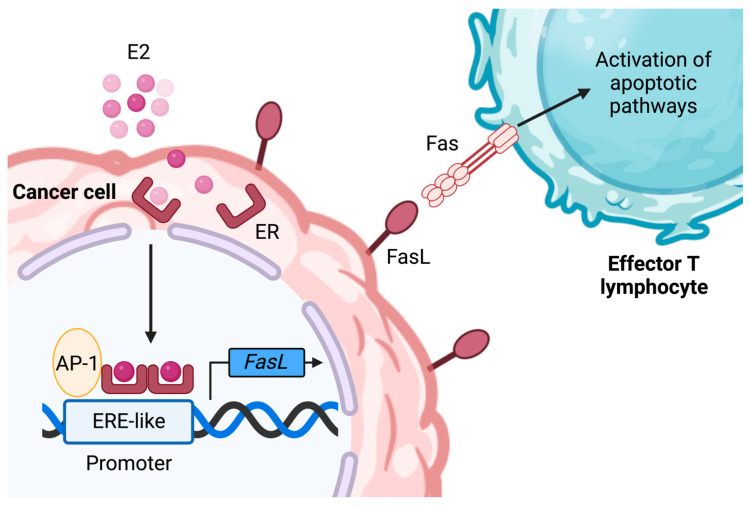
The impact of estrogen signaling through ER on the expression of FasL. In ER-positive MCF-7 and T47D cells, the association of the dimerized ER with AP-1 allows the transcription of the FasL gene. The interaction between FasL and Fas leads to the activation of apoptotic pathways in effector T lymphocytes, thereby resulting in anti-tumoral immune response impairment. E_2_, 17-β estradiol; ER, estrogen receptor; FasL, Fas ligand; AP-1, activating protein 1; ERE, estrogen response element. Illustration created using BioRender.

Breast tumor cells have also been shown to express Fas ligand (FasL). FasL induces the apoptosis of Fas-expressing activated lymphocytes [48,49]. It has been reported that the E_2_ treatment of ER^+^ BC cells MCF-7 and T47D increased Fas-L mRNA and protein expression. This effect can be inhibited by tamoxifen in a dose-dependent manner [49,50]. A closeup of the FasL gene reveals the presence of two motifs resembling EREs in the promoter region (including one with perfect homology), as well as the AP-1 enhancer element, which mediates yet another pathway for ER transcriptional regulation [49] (Figure 3). Altogether, this information advocates for the role of ER in the upregulation of FasL in BC cells as a mechanism of escape from CTL-mediated elimination.

### 3.2. The Link between ER and Immune Inhibitory Receptor Ligand PDL1 in BC

The immune inhibitory receptor ligand PDL1 has been reported to be upregulated in triple-negative and basal-like BC but not in luminal BC [51,52,53,54]. The binding of PDL1 to its receptor PD1, itself expressed by activated T lymphocytes, leads to their inhibition and thus the prevention of cancer cells killed by CTL [55,56]. Blocking anti-PD1 monoclonal antibodies (mAbs) have been developed and obtained FDA approval for the treatment of TNBC patients in the early and late stages of the disease, in combination with chemotherapy [13,14] (Figure 1). Importantly, because ER^+^ BC only sparsely expresses PDL1, the response of this category of patients to immune checkpoint inhibitor-based immunotherapy has been limited thus far.

**Figure 4:**
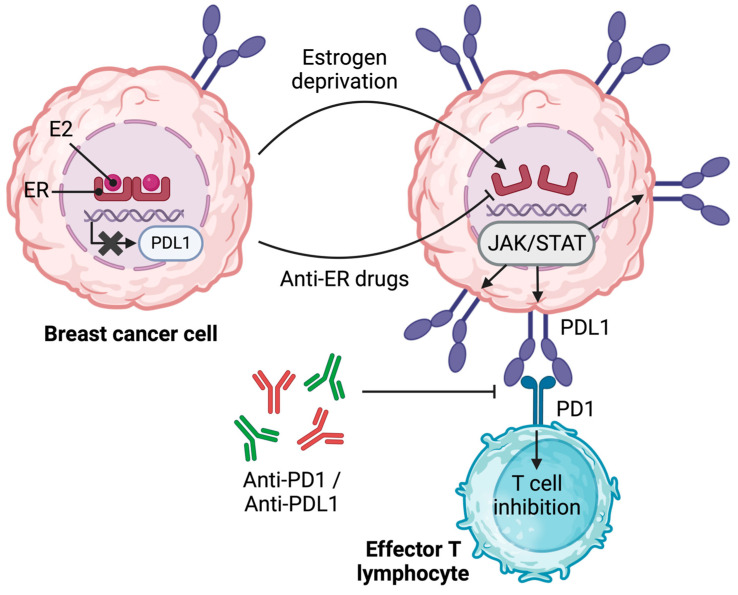
Estrogen-mediated regulation of PDL1 expression by breast cancer cells. Estrogen stimulation of breast cancer cells leads to the inhibition of PDL1 expression. Upon estrogen deprivation or ER antagonizing, signaling through JAK/STAT, and subsequently PDL1 expression, are promoted. The interaction between PD1 and PDL1 results in T cell inhibition. This interaction can then be disrupted by anti-PD1 or/and anti-PDL1 antibodies. E_2_, 17-β estradiol; ER, estrogen receptor; PDL1, programmed-death ligand 1; JAK, Janus kinase; STAT, signal transducer and activator of transcription; PD1, programmed cell death 1. Illustration created using BioRender.

Pre-clinical in vitro studies indicated that PDL1 expression was reduced in ERα^+^ (MCF-7, T47D, CAMA-1, ZR-75-1, and BT-474) compared to ERα^−^ (MDA-MB-231, HCC1937, and BT-549) BC cell lines, suggesting that ER signaling may lead to the downregulation of PDL1 (Figure 1). Consistently, the E_2_-triggering of ER has been found to restrain the expression of PDL1 proteins in MCF-7 but not in MDA-MB-231 BC cells, an effect that can be reversed by culturing the former in a steroid-free medium or fulvestrant treatment [30,57,58]. These observations have yet to be confirmed by others and extended to more BC cell lines. Interestingly, persistent estrogen deprivation also led to the activation of an immunosuppressive phenotype in MCF-7 cells, notably characterized by the secretion of IL-6 (an immunosuppressive cytokine negatively affecting immunotherapies) and the activation of JAK/STAT and NFκB signaling pathways (key regulators of PDL1 expression) [30,59] (Figure 4). These data highlight the importance of steroid hormones and their cognate receptors in the regulation of PDL1 expression in BC in vitro [30,57].

In a pre-clinical in vivo ER^+^ BC mouse model (MMTV-PyMT), tamoxifen was found to upregulate tumoral PDL1 [30]. Consistently, in the same study, an analysis of TCGA BC data highlighted an inverse correlation between ERα and PDL1 expression at the mRNA level [30]. Furthermore, the expression of PDL1 was evaluated in the primary tumor and matched metastases occurring during or after adjuvant anti-hormonal therapy in ER^+^ BC patients. PDL1 expression was low or undetectable in primary tumors but increased in half of the metastatic biopsies [30]. This observation further indicates that anti-ER drugs may increase the expression of PDL1 by BC cells, thereby potentially impairing anti-tumoral T cells (Figure 4). Along these lines, an analysis of transcriptomic data from the METABRIC cohort indicated an increased expression of genes related to several immune checkpoints, including *CD274* (PDL1), *PDCD1LG2* (PDL2), or *LGALS9* (galectin-9 was detected in tumors from patients receiving hormone therapy [30,51,60,61]). These results, therefore, argue in favor of a negative regulation of PDL1 by ER, which can be reversed by the ER-antagonizing action of anti-hormone therapies. Importantly, these observations suggest that anti-hormone therapy regimens, by increasing the BC cell expression of PDL1, may potentially enhance the efficacy of immunotherapies targeting the PD1/PDL1 axis (Figure 4), but combination approaches associating hormone therapies and anti-PD1/PDL1-based immunotherapies remain to be formally evaluated.

Mechanistically, the expression of PDL1 in various types of cancers can be regulated at the epigenetic level by promoter methylation and histone deacetylation [62]. In this context, the ten-eleven translocation 2 (TET2) DNA dioxygenase has been identified as an estrogen-regulated demethylase [63] regulating PDL1 expression in ER^+^ BC [63]. A low basal expression of PDL1 mRNA was detected by RNAseq analysis in MCF-7 BC cells compared to their TET2 KO counterparts in which PDL1 mRNA was upregulated [64]. This observation suggests that TET2 regulates PDL1 transcription in ER^+^ BC (Figure 1). Further supporting these findings, PDL1 is strongly expressed in ER^−^ BC cells, displaying low levels of TET2, such as the MDA-MB-231 cell line, as discussed earlier in this section. It is thus not surprising that TET2 overexpression in these BC cells correlates with the downregulation of PDL1 transcription [64]. Functionally, TET2 binds to the promoter region of the PDL1 gene and recruits HDAC1/2 histone deacetylases, a function that differs from its recognized role as a demethylating agent [64,65,66,67]. As a whole, the current state of knowledge in ER^+^ BC strongly suggests the involvement of the estrogen/ER axis in the regulation of PDL1, although characterization of the associated mechanisms requires further work.

### 3.3. ER Signaling and the Production of Immunosuppressive Factors by BC Cells

Additional mechanisms of cancer immune escape involve the generation of an immunosuppressive tumor environment, which reduces the efficacy of immunotherapies. Some of these mechanisms include enzymes such as indoleamine 2,3-dioxygenase (IDO), cyclooxygenase 2 (Cox2), adenosine, and immunosuppressive cytokines, such as TGFβ, IL-6 or IL-10 [2,3].

*ER signaling and IDO expression*

IDO, expressed by many cancers, including BC [68], is a key enzyme catalyzing the first, rate-limiting step of tryptophan degradation. Tryptophan deprivation, together with the production of immunosuppressive intermediary molecules, leads to T lymphocyte and NK cell inhibition [68]. IDO expression by BC cells remains to be clearly established, and its functional status in BC cells still needs to be unequivocally defined. Nevertheless, IHC analyses have indicated that IDO protein expression increased in malignant ductal cells found in ER^+^ compared to ER^−^ tumors [69]. These observations, however, contradict previous results, indicating that IDO mRNA (*INDO*) levels correlated with basal-like subtypes along with ER-negativity (Figure 1) [70]. In another study, lower Kyn serum concentrations (a byproduct of IDO1 activity) in ER^+^ patients at the time of diagnosis were correlated with low or absent IDO1 mRNA and protein expression in ER^+^ BC tissues, while the opposite was observed in ER^−^ tumors [71]. An RNAseq analysis of invasive breast cancer specimens from the TCGA highlighted a negative correlation between *IDO1* and *ESR1* gene expression, along with a significant downregulation of IDO, specifically in luminal A and B BC subtypes [71]. In an effort to uncover the molecular mechanisms underlying the BC subtype-dependent differential expression of IDO1, Dewi et al. analyzed CpG methylation patterns in the IDO1 promoter retrieved from whole genome bisulfite sequencing (WBGS) data as part of the TCGA [71]. The majority of studied CpG were found to be methylated in ER^+^ but not in ER^−^ BC samples, a finding that was further corroborated in vitro by a MassARRAY analysis of IDO1 promoter methylation patterns in ER^+^ BC cell lines (namely MCF-7, ZR-75-1, and BT-474), which contrasted with ER^−^ MDA-MB-231 BC cells [71].

*ER signaling and Cox2 expression*

Cox2 is another enzyme involved in the mechanisms underlying the immunosuppressive function of pro-tumoral immune cells and cancer cells themselves. Cox2 is overexpressed in a variety of solid cancers, and its activity leads to the inhibition of anti-tumoral immune cells [72,73]. If elevated, Cox2 mRNA and protein expression in BC have been reported in many reports, and very little is known about the impact of ER on the regulation of Cox2 expression/activity (Figure 1) [74,75,76,77]. In fact, very conflicting results have been obtained in anatomopathological studies concerning the potential impact of ER on Cox2 expression in BC tumors. While some reports have indicated that ER does not correlate with Cox2 expression [78,79,80], others have observed an inverse correlation between ER and Cox2 expression, associating this immunosuppressive enzyme with ER^−^ tumors [81,82,83]. More recent work also demonstrated that a high co-expression of Cox2 and nitric oxide synthase 2 (NOS2, another important immunosuppressive enzyme) was associated with poor survival of ER^−^ but not ER^+^ patients [84]. In this context, most studies related to Cox2 and NOS2 have been conducted in ER^−^ BC samples and pre-clinical models.

*ER signaling and adenosine production*

Adenosine plays a major immunosuppressive role within the TME through the inhibition of T lymphocytes and NK cell cytotoxic activity and infiltration in tumor beds [85]. Extracellular adenosine production results from the degradation of AMP (adenosine monophosphate) nucleotides by ecto-nucleotidases, particularly by 5′-nucleotidase (eN, commonly referred to as CD73) [85,86]. Extracellular adenosine production from AMP and ATP was found to be significantly increased in ER^−^ BC lines (MDA-MB-231, BT-549) compared to ER^+^ ones (ZR-75-1, MCF-7), which express CD73 at low to undetectable levels [86]. Low CD73 mRNA and protein levels were detected in MCF-7 cells treated with estradiol in a steroid-free medium, while tamoxifen or fulvestrant treatment led to the opposite effect [86]. These results argue for a negative regulation of CD73 by ERs in BC (Figure 1). The authors further hypothesized that the downregulation of CD73 observed in ER^+^ BC cell lines may be indirectly caused by ERs and may involve other actors, such as AP-1 and Sp1 transcription factors, which were shown to be implicated in CD73 promoter activity [87].

*ER signaling and immunosuppressive factor production*

Tumor-derived cytokines, such as IL-10 or TGF-β, represent other major mechanisms of cancer-induced immunosuppression. Although their role in breast cancers is well-studied, much less is known about their relationship to specific subtypes of BC, and the link between ER status and IL-10 expression has yet to be fully investigated. A study by Chavey et al. reported that IL-10 expression in BC specimens increased compared to healthy breast tissue and was inversely correlated to ER expression level [88]. In contrast, other studies reported that IL-10 expression was significantly higher in ER^+^ tumor specimens [89] (Figure 1).

Similarly, the specific interactions between ERα and TGF-β in BC are not entirely understood. Under physiological conditions, the two pathways are known to co-regulate one another to restrain mammary epithelial cell proliferation to homeostatic levels [90]. ER^+^ BC cells, such as MCF-7, are known to secrete biologically active forms of TGF-β, a production that can be positively modulated and enhanced following treatment with tamoxifen and 4-OHT [91]. These initial observations thus led to the hypothesis that TGF-β secretion and activation in BC cells could be regulated by estrogen signaling [91] (Figure 1). The secretion of TGF-β1 and TGF-β2 distinct isoforms by MCF-7 following exposure to tamoxifen were later evaluated. The antagonizing action of the ER modulator led to an increase in TGF-β2 mRNA and its secretion and was also associated with the activation of TGF-β1, most likely through the regulation of a protease involved in the conversion of TGF-β1 into its biologically active form [92]. It has been shown in one study that tamoxifen- or fulvestrant-induced TGF-β production by BC cells (primarily MCF-7) resulted in the inhibition of immune effector cytotoxic mechanisms specific to CD8^+^ T lymphocytes (the production of granzyme B, perforin, and FasL), which was associated with an increase in the tumor-promoting immunosuppressive regulatory T cells (T_reg_) pool [93]. Estrogen signaling seems to downregulate TGF-β production by ER^+^ BC cells (Figure 1). Although these preliminary observations remain limited, as they focus on one cell line, they open the door to prospective pre-clinical and clinical studies that aim at decipher the role of ERs on the production of immunosuppressive cytokines in BC.

## 4. The Effects of ER Signaling and the Impact of ER Modulation on Immune Cell Function in the Context of BC

Apart from its direct impact on BC cells, the role of the ER signaling pathway in cells of the TIME represents an active area of research. Relevant to the current topic, some studies have suggested that different immune cell subsets can respond to estrogens and, in turn, participate in BC progression [8,94,95,96,97,98,99,100]. In this context, estrogens have been recognized as important immune modulators [101]. However, to date, data related to the direct impact of ER signaling and its modulation on immune cells remain relatively sparse. In this section, we review and discuss the state of current knowledge related to this problem and highlight the remaining questions in the field.

### 4.1. ER Signaling in the Immune Cells of the Myeloid Lineage

Myeloid cells encompass highly heterogeneous subpopulations, play a variety of roles in BC, and are considered the main drivers of tumor progression [12,102,103,104]. Recent studies have reported that estrogen signaling contributes to the dysregulation of myelopoiesis observed in cancers [100]. Although most reports are not specific to BC, the conclusions drawn from these studies hint at common mechanisms across cancer types. In a pre-clinical model of oöphorectomized ID8-*Defb29/Vegfa* ER^−^ ovarian cancer, myeloid-derived suppressor cells (MDSCs) were found to express ERα (Figure 1). Their recruitment to the spleen and tumor sites was significantly enhanced following E_2_ stimulation, resulting in accelerated tumor progression (Figure 1) [100]. E_2_ supplementation also enhanced the immunosuppressive activity of the granulocytic subset of MDSC (G-MDSC), an effect that was prevented following ER antagonizing by methylpiperidinopyrazole (MPP) [100]. Such effects were found to be mediated by ERα signaling through the upregulation of STAT3 phosphorylation in MDSCs [100] (Figure 1). In this same mouse model, the tumors of ERα^−/−^ KO animals reconstituted with ERα-deficient bone marrow failed to exhibit the progression kinetics of animals reconstituted with WT bone marrow, confirming a hematopoietic ERα signaling-dependent mechanism of cancer progression [100]. In agreement with these results, the treatment of ex vivo generated MDSCs from the bone marrow of lung cancer patients with MPP impaired their expansion and increased their maturation [100]. In the context of BC, it has been reported that E_2_ promoted the expansion of MDSCs generated ex vivo from patient bone marrow cells, while the ER dowregulators fulvestrant and JD128 conversely dampened this expansion when MDSCs were cultured in an E_2_-containing medium [24]. JD128 negatively impacted the phosphorylation and activation of STAT3 in ex vivo generated G-MDSCs, which reinforces previous observations by Svoronos et al. [24,100]. The treatment of the estrogen-insensitive 4T1 syngeneic TNBC model with fulvestrant or JD128 in the presence of anti-PDL1 antibodies resulted in tumor growth suppression, a significant reduction in MDSC numbers, and increased numbers of anti-tumoral CD4^+^ and CD8^+^ T lymphocytes [24]. Altogether, these results indicate that apart from a direct effect on BC cells, the estrogen signaling regulates the expansion and function of immune cells, specifically tumor-promoting MDSCs, suggesting that anti-hormone therapies may synergize with and enhance the efficacy of immunotherapies, even potentially in ER^−^ breast cancers (Figure 1).

Although MDSCs represent an important proportion of myeloid cells within the TME, other cell types such as tumor-associated macrophages (TAMs), dendritic cells (DCs), and monocytes can be impacted by estrogen signaling [105,106]. An analysis of macrophage polarization using CIBERSORT signatures on transcriptomic data sets emanating from melanoma patients treated with immune checkpoint blockade (anti-PD1 and anti-CTLA4) demonstrated that TAMs, and more specifically the M1/M2 ratio, are reliable markers to predict the response to this class of immunotherapies [107,108]. Interestingly, in the fraction of non-responder patients, the expression of the *CYP19A1* enzyme was found to positively correlate with the presence of TAMs [107,108], hinting at a potential link between estrogen signaling and the polarization of macrophages toward a pro-tumorigenic, M2-type phenotype (Figure 1). A functional assessment of the impact of E_2_ in ovariectomized syngeneic melanoma models (B16F10, YuMM5.1, and BPD6) further confirms this in silico data and demonstrates the E_2_-dependent recruitment of monocytes to the tumor beds and a skewing of the M1/M2 ratio in favor of the latter, leading to an enhanced immunosuppressive TME and the facilitation of melanoma tumor growth (Figure 1) [108]. In these pre-clinical models, treatment with fulvestrant reversed the effects induced by E_2_ supplementation and enhanced the response to ICB (anti-PD1 and anti-CTLA4), indicating the potential of this ER downregulator as a TME remodeling agent [108]. Future studies are clearly needed to evaluate whether similar effects may also be extended to BC, opening new potential therapeutic applications for ER^−^ BC.

### 4.2. ER Signaling in Immune Cells of the Lymphoid Lineage

To date, most of the studies exploring the impact of estrogens on lymphoid cell functions have been conducted in pathologies other than cancers. Nevertheless, they set a strong basis for such explorations in the near future. Generally, estrogens exert dual roles on immune cells depending on the context. While low, physiological doses of estrogens have been associated with pro-inflammatory T_h_1 responses, and higher estrogen concentrations seem to promote T_h_2, anti-inflammatory immunity [109,110]. Pregnancy levels of E_2_ were found to enhance IL-10 and IFNγ production and, at the same time, inhibit tumor-necrosis factor α (TNF-α) secretion by CD4^+^ T lymphocytes [111,112,113,114]. This finding is particularly relevant, as BCs are associated with increased levels of estrogens [115]. As reported by Polanczyk et al., high levels of estrogens, consistent with those associated with pregnancy, play an important role in the expansion and function of immunosuppressive T_regs_, notably by conferring them with the expression of PD1 (Figure 1) [116,117,118]. In this context, estrogen levels exceeding physiological concentrations in ovariectomized mice were also found to decrease the cytotoxic potential of NK cells through a decrease in the production of TNF-α and granzyme B [119], an observation that was extended to humans [120]. In B cells, high levels of E_2_ were also associated with an auto-immune potential, which is characterized by an increased production of antibodies [121]. Although these studies do not examine the impact of the estrogen/ER axis in cancer specifically, the above results may hint at similar regulation mechanisms existing in the context of BC and advocate for the need for future studies to clearly delineate the role and impact of ER signaling in immune cells in the setting of BC.

## 5. Conclusions and Perspectives

To date, and despite the development of innovative treatments, BC remains the leading cause of cancer-related death in women [122]. Although the majority of BCs are estrogen-dependent and impacted patients benefit from anti-hormone therapy, resistance to treatment may develop over time, and relapse frequently occurs [123]. If other subtypes of BC, such as triple-negative or basal-like, may benefit from the advent of immunotherapies in certain contexts, ER^+^ BC has yet to experience such breakthroughs [124]. The main reason behind the limited efficacy of immunotherapies in ER^+^ BC lies in its low immunogenicity and the triggering of multiple mechanisms of immune escape, including suppressive immune cell subsets, such as MDSCs and T_regs_. As outlined above, many studies have attempted to address the impact of ER signaling on the resistance/sensitivity of BC cells to the mechanisms of protective anti-tumor immunity and have highlighted that interfering with ER signaling may enhance tumor elimination by immune effectors (Table 1). These observations are, however, for the most part, correlative, and warrant future studies to accurately decipher the mechanistic bases underlying the effects of ER signaling on BC cell resistance to immune elimination and BC cell-induced immunosuppression pathways. More specifically, important interrogations remain as to which ER isoforms may mediate the observed effects and, likewise, the distinction between genomic and non-genomic signaling pathways is not always established. This is especially relevant since HR^−^ BCs, such as triple-negative or basal-like ones, may exhibit sensitivity to estrogens through non-genomic circuits involving the G-protein coupled estrogen receptor (GPER) [125,126]. Similarly, a better understanding of the putative direct immunomodulatory effects of ER signaling and its impact on myeloid and lymphoid cells involved in tumor immunity and tumor immune escape is required. These prospective studies may further highlight the potential of using hormone-based therapeutic approaches to improve responses to immunotherapies and may open the door for the design and development of hormono-immunotherapeutic combination modalities extended to ER^−^ BC subtypes.

## Figures and Tables

**Table 1:** Summary of the pre-clinical and clinical observations of cancer-intrinsic resistance mechanisms to anti-tumoral immune mechanisms in the context of breast cancers.

Molecular Actor Investigated	*Pre-Clinical Observations*		Clinical Observations	References
	In Vitro	In Vivo		
MHC I (HLA-I) presentation	HLA-I expression is enhanced by E_2_ in MCF-7 (ER^+^) but not in MDA-MB-231/MDA-MB-435s (ER^−^) Inverse correlation between ER status and HLA-A,B,C gene expression in BC cell lines ER antagonizing increases HLA-A,B,C proteins in ER^+^ MCF-7 and T47D BC cell lines	No studies performed to date	HLA-A,B,C genes negatively correlate with ER in treatment-naïve patients with primary BC HLA-A,B,C increased following treatment with estrogen modulators (tamoxifen, goserelin)	[23,24,25]
β-2- microglobulin	No studies performed to date		Reduced protein expression in ER^+^ patients; no difference of expression at the mRNA level	[27]
TAP1/2	No studies performed to date		No clear association with ER status	[36,37,38]
PI-9	E_2_ and tamoxifen are inducers of the protease, while raloxifene and fulvestrant block its production in MCF-7 cells MCF-7 CSC expresses a higher level of PI-9	No studies performed to date	No studies performed to date	[42,43]
FasL	E_2_ increases FasL mRNA in MCF-7 and T47D cells; tamoxifen decreases it	No studies performed to date	No studies performed to date	[45,46]
Immune checkpoint molecules	PDL1 protein is downregulated in ER^+^ BC cells lines (MCF-7, T47D, CAMA-1, ZR-75-1, BT-474) but not ER^−^ ones (MDA-MB-231, HCC1937, BT-549)	Tamoxifen treatment of tumor-bearing MMTV-PyMT upregulates tumor PDL1 expression	PDL1 mRNA inversely correlates with ERα (TCGA) Patients treated with anti-hormonal therapy have an increased mRNA expression of PDL1, PDL1, LGALS9, CD86, and CD48	[26,47,53,54,56,57]
IDO	Prominent methylation of the IDO1 promoter in ER^+^ (MCF-7, ZR-75-1, BT-474) compared to ER^−^ (MDA-MB-231) BC cells	No studies performed to date	One study shows increased IDO protein in ER^+^ BC [65] IDO mRNA is found in higher quantities in ER^−^ BC specimens; there is a negative correlation between *ESR1* and *IDO1*	[65,66,67]
Cox2 NOS2	No studies performed to date	No studies performed to date	Contradictory observations have been reported	[74,75,76,77,78,79]
CD73 (eN)	Low to undetectable levels in ER^+^ BC cells (MCF-7, ZR-75-1), which is reversed by tamoxifen or fulvestrant treatment High activity of the enzyme in ER^−^ BC cells (MDA-MD-231, BT-549)	No studies performed to date	No studies performed to date	[82,83]
Immunosuppressive cytokines	No studies performed to date	No studies performed to date	Contradictory observations have been reported	[84,85]
	TGF-β secretion by MCF-7 is enhanced following tamoxifen and fulvestrant treatment	No studies performed to date	No studies performed to date	[88,89]
